# A Boom in Nanotechnologies for a High Level of Precision Medicine

**DOI:** 10.3390/cancers15092522

**Published:** 2023-04-28

**Authors:** Eurydice Angeli, Guilhem Bousquet

**Affiliations:** 1 Paris Cité University, INSERM, UMR_S942 MASCOT, F-75006 Paris, France; eurydice.angeli@gmail.com; 2 Institut Bergonié, Department of Medical Oncology, F-33000 Bordeaux, France; 3 Sorbonne Paris Nord University, 99 Avenue Jean Baptiste Clément, F-93430 Villetaneuse, France; 4 Department of Medical Oncology, APHP, Avicenne Hospital, F-93000 Bobigny, France

The number of publications on nanomedicine in oncology has been exponential over the last ten years, going from 640 publications in 2012 to 2487 publications in 2022, reflecting the growing interest and potential of these new technologies.

Since observations made in the 1970s and 1980s regarding the preferential extravasation of nano-encapsulated drugs in tumors, the enhanced permeability and retention (EPR) effect [1,2] has been identified, and it founded the rationale of nanomedicines. Parallel advances in the knowledge of cancer cell molecular and metabolism hallmarks considerably improved the conceptional design of nanotechnologies for cancer detection and treatment, allowing them to achieve a high level of precision medicine.

Technological progress of nanoparticles enabled the successful development of molecules such as mRNA/siRNA, whose therapeutic concept has emerged since the 1990s [3]. Thus, the COVID-19 pandemic has exposed 10 years of nanoparticle research, with the development of nano-formulated mRNA vaccines that have been safely administered to thousands of people around the world. In oncology, more than 15 molecules have been approved by the FDA, and to date, there are more than 20 molecules in development [4].

This Special Issue exposes a wide range of applications of nanoparticles in cancer area, particularly for therapeutic applications where they exceed conventional therapies by their better pharmacological profile [5]. Innovative formulations allow for a longer lifespan in the body, as well as better tumor targeting [4,5,6,7]. Thanks to their encapsulation, they have the ability to bypass physiological barriers and can be modeled as desired [8]. They benefit from particular physicochemical properties of inorganic nanoparticles, such as nanocrystals (energy absorption and heat release of irradiated gold nanoparticles, fluorescence emission of quantum dots) [8,9]. They can have their own cytotoxic anti-tumor effect via the release of pro-apoptotic reactive oxygen species [7,8,9], or through iron deprivation [6]. They can also induce indirect cytotoxicity via an immuno-stimulating effect [6,7]. As diagnostic tools, they have excellent qualities of sensitivity and specificity [6,8]. Due to their magnetic properties, iron nanoparticles could replace the use of contrast media in MRI [8]. Nanoparticles can also be used as tools for modeling physical phenomena, such as the mechanical compression performed on tissues by prosthetic material [10].

Thanks to improved molecular and histological screening techniques, nanoparticles are emerging as ideal candidates for personalized medicine, opening broad prospects for pharmaceutical research and drug development. This is evidenced by the current boom in the development of nano-production platforms.

Despite the rise in these new technologies, several challenges remain.

Concerning the pharmacological knowledge of nanoparticles in the circulating phase, a meta-analysis on preclinical models revealed that after systemic injection, only 0.7% of nanoparticles accumulated at the cancer site [11]. Indeed, targeting ligands may be hidden under the protein corona, a layer of proteins that binds to the surface of the nanoparticle in the biological medium, and can change their biodistribution. Furthermore, the protein corona can change the surface charge and hydrodynamic size of the nanoparticles, modifying their characteristics [12]. Once in the cytosol, nanomedicines can be sequestered in intracellular vesicles such as lysosomes, where their cargo can be degraded or lost to the vesicle recycling [13]. Excessive stability of the formulation can also compromise drug release and hence its bioavailability [13].

Concerning strategies for circumventing the tumor barrier, despite abundant data on intratumor accumulation by the EPR effect, there is a great heterogeneity in the distribution of nanoparticles in the tumor and tumor microenvironment [14]. The tumor microenvironment limits the effectiveness of drugs by preventing their accumulation, distribution and inhibiting the immune response. Several strategies for “normalizing” the tumor microenvironment are being studied to restore the effective penetration of nanoparticles within [15]. The future will probably be the association of these multiple targets within the same nanoparticle.

The complex molecular assembly of nanoparticles makes them highly specific; thus, this technology is part of a personalized medicine strategy. We will, therefore, need to develop more biomarkers predictive of efficiency, integrating big data and artificial intelligence to optimize design of nanoparticles by building predictive models of their biodistribution, uptake and other biological outcomes.
